# Blacks’ Diminished Returns of Parental Education on Household Income: A Study of College Students in the US

**DOI:** 10.31586/ojer.2024.1016

**Published:** 2024-08-04

**Authors:** Shervin Assari, Payam Sheikhattari, Hossein Zare

**Affiliations:** 1 Department of Internal Medicine, Charles R. Drew University of Medicine and Science, Los Angeles, CA, United States; 2 Department of Family Medicine, Charles R. Drew University of Medicine and Science, Los Angeles, CA, United States; 3 Department of Urban Public Health, Charles R. Drew University of Medicine and Science, Los Angeles, CA, United States; 4 Marginalization-Related Diminished Returns (MDRs) Center, Los Angeles, CA, United States; 5 Center for Urban Health Disparities Research and Innovation, Morgan State University, Baltimore, MD, USA; 6 The Prevention Sciences Research Center, School of Community Health and Policy, Morgan State University, Baltimore, MD, USA; 7 Department of Behavioral Health Science, School of Community Health and Policy, Morgan State University, Baltimore, MD, USA; 8 Department of Health Policy and Management, Johns Hopkins Bloomberg School of Public Health, Baltimore, MD, United States; 9 School of Business, University of Maryland Global Campus (UMGC), Adelphi, MD, United States

**Keywords:** Racism, Ethnic Groups, Social Determinants of Health

## Abstract

**Background:**

Parental education is a key determinant of household income, but its benefits are not uniformly distributed across racial groups. According to the Minorities’ Diminished Returns (MDRs) theory, the socioeconomic benefits of resources such as parental education are systematically lower for minority families, particularly Blacks who have been subjected to slavery, segregation, racism, and discrimination.

**Objective:**

This study aims to investigate the diminished returns of parental education on household income among Black college students in the US.

**Methods:**

Using data from the Higher Education Research Institute (HERI) Freshman Student Survey, we analyzed the associations between race, parental education, and household income. The sample included 2,235,733 students, comprising 2,191,543 White and 441,90 Black freshman college students. We conducted regression analyses to examine the association between parental education and household income, adjusting for relevant covariates.

**Results:**

Our findings indicated that higher parental education is associated with higher household income in the pooled sample. We also found a positive association between parental education and household income for both Black and White college students. However, the magnitude of this positive association was significantly smaller for Black students compared to White students, demonstrating diminished returns of parental education on household income for Black families.

**Discussion:**

The results support the theory of Minorities’ Diminished Returns, highlighting the need for policies that address the systemic barriers contributing to sustained economic inequality. These barriers go beyond parental education, resulting in income differences between similarly educated White and Black families.

## 1. Introduction

Parental education has long been recognized as a major driver of household income, serving as a key determinant of a family’s socioeconomic status [1,2]. For many individuals, including working parents, higher levels of educational attainment translate into better job opportunities, higher earnings, and greater economic stability for their families [3–5]. Consequently, parental educational attainment and household income are closely correlated, and both indicators are sometimes used interchangeably as measures of socioeconomic status (SES) [6–8].

However, the benefits of educational attainment are not uniformly distributed across different racial groups [9,10]. Research in the US has shown that the effects of educational attainment on household and individual-level income [11], as well as wealth [12], can vary significantly by race. Black individuals often experience significantly lower returns on their educational investments compared to their White counterparts [13]. This suggests that the interchangeability of various SES indicators may not be consistent across racial groups due to differential correlations by race [14–20].

This differential effect can be attributed to various factors, including the quality of education received and labor market discrimination [21–23]. Black individuals are more likely to attend under-resourced schools, which can limit their educational attainment and subsequent economic opportunities [24–26]. Additionally, systemic discrimination in the labor market can result in lower wages and fewer job opportunities for equally qualified Black individuals compared to their White peers [21–23].

These disparities align with the theory of Minorities’ Diminished Returns (MDRs) [27,28], which posits that the benefits of one socioeconomic resource, such as educational attainment, on other resources, such as family income, are systematically lower for minoritized and racialized groups compared to White families and individuals [11,29]. Numerous studies have provided evidence supporting MDRs across various outcomes, including health, employment, and economic stability [12].

As a result of these diminished returns, when studies compare Black and White individuals for the developmental and health effects of parental education, we observe weaker effects for Black children and youth [30]. These diminished returns manifest in various domains, including educational performance, cognitive development, behavioral outcomes, and overall health. For instance, higher parental education tends to be associated with better academic achievement and cognitive skills in White children, but these benefits are significantly attenuated for Black children [31–33]. Similarly, the protective effects of parental education on behavioral problems and mental health are stronger for White youth than for their Black counterparts. This consistent pattern highlights the pervasive impact of structural inequalities and systemic racism, which undermine the potential benefits of parental education for Black families [30,34,35].

However, there is a gap in the literature regarding the diminished returns of parental education on household income specifically for college students in the US [36–38]. Understanding this phenomenon is crucial, as it can shed light on the broader systemic issues that contribute to economic inequality.

The aim of this study is to test the diminished returns of parental education on household income among college students in the US, using data from the Higher Education Research Institute (HERI) Freshman Student Survey [39–41]. We hypothesized a strong positive correlation between parental education and household income in the pooled sample. At the same time, we expected a weaker association between parental education and household income for Black families compared to White families, due to factors such as racism, stratification, and segregation.

## 2. Methods

### 2.1. Design and Setting

This study is cross-sectional and utilizes data from the American Freshman surveys spanning from 1966 to 2019. The American Freshman surveys, conducted annually, capture over 40 years of insights into the characteristics, attitudes, values, educational achievements, and aspirations of college students in the United States. Initiated by the Cooperative Institutional Research Program (CIRP) every fall since 1966, these surveys provide a comprehensive view of the evolving nature of American college students [42– 44].

These annual surveys reflect not only changes within higher education but also broader societal shifts. This report offers an overview of the first twenty-five years of CIRP data, highlighting significant findings and their implications for both American higher education and society. The initial seven surveys were conducted by the American Council on Education, with funding from the Carnegie Corporation of New York and the Ford Foundation. Since 1972, the Higher Education Research Institute (HERI) at the University of California, Los Angeles, has taken over the annual CIRP freshman surveys, with continued support from the American Council on Education [40].

Each year, CIRP surveys approximately 250,000 full-time students from around 600 two- and four-year colleges and universities across the nation. The HERI data covers eight broad topics: academic skills and preparation, demographic trends, high school activities and experiences, educational and career plans, majors and careers, attitudes, student values, and financing college [43].

### 2.2. Measures

Variables in the current analysis were race, ethnicity, age, sex, survey year, parental education, and belief/attitude about discrimination. Race, ethnicity, age, sex, parental education, and belief/attitude about discrimination were all self-reported. Race was self- identified and was Black and White. Ethnicity was Latino vs. Non-Latino. Age was an ordinal variable ranging from 1 to 11: 16 or younger, 17, 18, 19, 20, 21 or older, 22 to 24, 25 to 29, 30 to 39, and 40 to 54. Parental education was an ordinal variable ranging from 1 to 8. 1: Junior High, 2: Some High School, 3: High School Graduate, 4: Post Secondary School Other than College, 5: Some College, 6: College Degree, 7: Some Graduate, and 8: Graduate Degree. Male was coded one and female was coded zero for sex. Outcome was self-reported household income: This variable included the following 25 levels: Less than $6,000, $6000–$9999, Less than $10,000, $10,000–$14,999, $15,000–$19,999, $20,000–$24,999, $25,000–$29,999, $30,000 or more, $30,000–$34,999, $30,000–$39,999, $35,000–$39,999, $40,000 or more, $40,000–$49,999, $50,000 or more, $50,000–$59,999, $50,000–$99,999, $60,000–$74,999, $75,000–$99,999, $100,000 or more, $100,000–$149,999, $150,000 or more, $150,000–$199,999, $200,000 or more, $200,000–$249,999, and $250,000 or more. As income was measured across different years, we calculated CPI-adjusted income to ensure comparability across all years. The Consumer Price Index (CPI) [45–48] reflects inflation and changes in the value of money over time, making it a crucial tool for adjusting income data collected over multiple years. By adjusting for CPI, we converted all income values to constant dollars, which allows us to compare income levels from different years on a consistent scale, free from the distortions caused by inflation. This adjustment ensures that any observed differences in income are due to actual changes in economic status rather than inflationary effects. Therefore, our analysis accurately reflects the real value of income and provides a more precise understanding of the relationship between parental education and household income over time.

### 2.3. Statistical Analysis

For the descriptive analysis, we calculated frequencies and percentages to summarize the distribution of the key variables, including race, parental education, age, sex, and year of survey. For multivariable analysis, we conducted linear regression models to examine the relationship between parental education and household income, with race as a moderator and age, sex, and year of survey as covariates. *Model 1* included the main effects of parental education and race, along with the confounders. *Model 2* added an interaction term between race and parental education to test whether the effect of parental education on household income varied by race. *Model 3* was performed in White and *Model 4* was performed in Black sample. From both models, we reported unstandardized b, standardized beta coefficients (β), 95% confidence intervals (CIs), a p values to quantify the strength of the associations. All statistical analyses were performed using SPSS software, and a significance level of 0.05 was used to determine statistical significance.

## 3. Results

The sample included 2,235,733 students, comprising 2,191,543 White and 44190 Black freshman college students. Table 1 presents a summary of the descriptive data of the pooled sample that included both Black and White participants. As this Table shows, most participants were White, and were around the age of 20.

As shown in Table 2, parental education was positively associated with family income (main effect b = 2.528).

As shown in Table 3, the positive association between parental education and family income were different for Black and White students, with Black students showing a weaker correlation than White students (interaction b =−.427).

As shown in Table 4, the positive association between parental education and family income were significant for Black (b =2.157) and White (b =2.531) students, with Black students showing a weaker correlation than White students.

## 4. Discussion

This study aimed to explore the diminished returns of parental educational attainment on household income among Black college students in the US, compared to their White counterparts. Our findings confirmed that while higher parental educational attainment correlates with increased household income for the pooled sample as well as both Black and White college students, the strength of this positive relationship is notably weaker for Black than White college students, indicating Blacks’ diminished returns of education on income.

Previous research has demonstrated MDRs across various domains, including health outcomes, employment opportunities, and economic stability. For example, studies have shown that Black individuals receive fewer health benefits from socioeconomic resources compared to White [49–51]. Similarly, higher educational attainment does not translate into equivalent employment opportunities for Black individuals, further perpetuating economic disparities. The concept of Minorities’ Diminished Returns (MDRs) [27,28] explains the observed disparities in our study. Several mechanisms may contribute to these diminished returns, including systemic racism, lower quality of education in predominantly Black schools, and labor market discrimination. These factors collectively hinder the economic benefits that Black individuals can derive from their parents’ educational achievements [30,35,52,53].

As a result of these diminished returns, when studies compare Black and White individuals for the developmental and health effects of parental education [49–51], we observe weaker effects for Black children and youth [35]. These diminished returns manifest in various domains, including educational performance, cognitive development, behavioral outcomes, and overall health. For instance, higher parental education tends to be associated with better academic achievement and cognitive skills in White children, but these benefits are significantly attenuated for Black children [54,55]. Similarly, the protective effects of parental education on behavioral problems and mental health are stronger for White youth than for their Black counterparts [56,57]. This consistent pattern highlights the pervasive impact of structural inequalities and systemic racism, which undermine the potential benefits of parental education for Black families [27].

### 4.1. Implications

The findings of this study have significant implications for policymakers and educators. There is a need for targeted interventions that address the systemic barriers contributing to diminished returns for Black individuals. This includes improving the quality of education in under-resourced schools, implementing anti-discrimination policies in the labor market, and providing additional support to Black students and their families to ensure that they can fully benefit from educational achievements.

### 4.2. Limitations

This study has several limitations. First, the cross-sectional design limits our ability to establish causality. Second, the data is self-reported, which may introduce reporting bias. Third, the sample may not be representative of all college students in the US, limiting the generalizability of the findings. In addition, the sample size was imbalanced, and we had more White than Black college students in our sample. Finally, all indicators in this study were individual and family level, and we did not include neighborhood factors, policies, or other higher-level indicators that could change the association between parental educational attainment and income. Future research should use longitudinal data to better understand the causal pathways and consider more diverse samples.

## 5. Conclusions

Our study provides further evidence of the diminished returns of parental educational attainment on household income among Blacks in the US. These findings, for the first time, show this phenomenon for a national sample of freshman college students in the US. These findings underscore the importance of addressing the systemic factors that contribute to economic inequality. By understanding and mitigating the barriers that lead to diminished returns, we can work towards a more equitable society where the benefits of education are accessible to all.

## Figures and Tables

**Table 1.** Descriptive data of White and Black students

	White		Black	
	n	%	n	%
**Gender**				
Female	1222726	55.8	26401	59.7
Male	968817	44.2	17789	40.3
**Age**				
16 or younger	4309	0.2	132	0.3
17	94138	4.3	1782	4.0
18	1416862	64.7	30609	69.3
19	444924	20.3	9062	20.5
20	88084	4.0	1130	2.6
21 or older	53630	2.4	294	0.7
22 to 24	60671	2.8	813	1.8
25 to 29	15879	0.7	198	0.4
30 to 39	9735	0.4	79	0.2
40 to 54	2960	0.1	28	0.1
16 or younger	351	0.0	63	0.1
**Year**				
1966	16219	0.7	0	0.0
1967	21310	1.0	0	0.0
1968	23100	1.1	0	0
1969	20929	1.0	0	0
1970	22366	1.0	0	0
1971	29941	1.4	199	0.5
1972	35081	1.6	297	0.7
1973	36637	1.7	216	0.5
1974	38688	1.8	295	0.7
1975	39765	1.8	374	0.8
1976	40804	1.9	248	0.6
1977	38869	1.8	240	0.5
1978	37271	1.7	266	0.6
1979	38492	1.8	275	0.6
1980	37565	1.7	256	0.6
1981	34917	1.6	252	0.6
1982	29432	1.3	291	0.7
1983	27413	1.3	267	0.6
1984	33522	1.5	289	0.7
1985	32795	1.5	390	0.9
1986	35027	1.6	381	0.9
1987	37124	1.7	519	1.2
1988	45626	2.1	559	1.3
1989	43712	2.0	577	1.3
1990	43962	2.0	485	1.1
1991	49899	2.3	665	1.5
1992	50567	2.3	718	1.6
1993	51879	2.4	819	1.9
1994	58555	2.7	1094	2.5
1995	54497	2.5	1135	2.6
1996	59523	2.7	1332	3.0
1997	60116	2.7	1764	4.0
1998	66694	3.0	1751	4.0
1999	69574	3.2	1878	4.2
2000	79873	3.6	2108	4.8
2001	83830	3.8	1433	3.2
2002	81941	3.7	1543	3.5
2003	78496	3.6	1705	3.9
2004	84811	3.9	2674	6.1
2005	74095	3.4	2361	5.3
2006	76242	3.5	3064	6.9
2007	74098	3.4	2941	6.7
2008	72594	3.3	2884	6.5
2009	62878	2.9	2735	6.2
2010	60814	2.8	2910	6.6
**Parental Education**				
1: Junior High	150490	6.9	1053	2.4
2: Some High School	211102	9.6	1228	2.8
3: High School Graduate	423434	19.3	5514	12.5
4: Post Secondary School Other than College	76944	3.5	1495	3.4
5: Some College	349976	16.0	8557	19.4
6: College Degree	460320	21.0	11702	26.5
7: Some Graduate	55121	2.5	1839	4.2
8: Graduate Degree	464156	21.2	12802	29.0
**Income (Unadjusted for CPI)**				
Less than $6,000	246064	11.2	1514	3.4
$6000–$9999	171707	7.8	996	2.3
Less than $10,000	54926	2.5	1547	3.5
$10,000–$14,999	224835	10.3	2411	5.5
$15,000–$19,999	149900	6.8	1988	4.5
$20,000–$24,999	156799	7.2	2688	6.1
$25,000–$29,999	123483	5.6	2436	5.5
$30,000 or more	1933	0.1	0	0.0
$30,000–$34,999	37768	1.7	465	1.1
$30,000–$39,999	140926	6.4	3745	8.5
$35,000–$39,999	29732	1.4	406	0.9
$40,000 or more	594	0.0	15	0.0
$40,000–$49,999	160385	7.3	4038	9.1
$50,000 or more	4894	0.2	154	0.3
$50,000–$59,999	132825	6.1	3691	8.4
$50,000–$99,999	12122	0.6	164	0.4
$60,000–$74,999	144823	6.6	4554	10.3
$75,000–$99,999’	138808	6.3	4624	10.5
$100,000 or more	4154	0.2	102	0.2
$100,000–$149,999	129858	5.9	4334	9.8
$150,000 or more	8261	0.4	208	0.5
$150,000–$199,999	47455	2.2	1704	3.9
$200,000 or more	27239	1.2	671	1.5
$200,000–$249,999	14908	0.7	605	1.4
$250,000 or more	27144	1.2	1130	2.6

**Table 2.** Logistic regression in the pooled sample (no interaction)

	Unstandardized Coefficients	Standardized Coefficients	95.0% Confidence Interval for B		Sig.
Race (Black)	.765	.050	.668	.863	< .001
Survey Year	−.085	.001	−.086	−.083	< .001
Male	1.274	.014	1.246	1.301	< .001
Age (1–11)	−.679	.006	−.691	−.667	< .001
Parental Education (1–8)	2.528	.003	2.521	2.534	< .001
Constant	175.756	1.154	173.493	178.019	< .001

**Table 3.** Logistic regression in the pooled sample (with interaction)

	Unstandardized Coefficients	Standardized Coefficients	95.0% Confidence Interval for B		Sig.
Race (Black)	3.217	.157	2.910	3.525	< .001
Survey Year	−.085	.001	−.086	−.084	< .001
Male	1.273	.014	1.246	1.300	< .001
Age (1–11)	−.678	.006	−.691	−.666	< .001
Parental Education (1–8)	2.534	.003	2.528	2.541	< .001
Parental Education (1–8) x Black	−.427	.026	−.477	−.376	< .001
Constant	176.197	1.155	173.934	178.460	< .001

**Table 4.** Logistic regression in the White and Black sample (stratified)

	Unstandardized Coefficients	Standardized Coefficients	95.0% Confidence Interval for B		Sig.
White					
Survey Year	−.083	.001	−.084	−.082	< .001
Male	1.262	.014	1.234	1.290	< .001
Age (1–11)	−.682	.006	−.695	−.670	< .001
Parental Education (1–8)	2.531	.003	2.525	2.538	< .001
Constant	171.957	1.162	169.679	174.235	< .001
Black					
Survey Year	−.264	.005	−.274	−.254	< .001
Male	1.786	.096	1.597	1.975	< .001
Age (1–11)	−.453	.050	−.551	−.354	< .001
Parental Education (1–8)	2.157	.025	2.108	2.206	< .001
Constant	536.384	10.260	516.274	556.495	< .001

## Data Availability

HERI data are available to public at https://heri.ucla.edu/.
